# The Factors Associated With Daily Life Support for Single Older Adults to Remain at Their Home

**DOI:** 10.1093/geroni/igaa057.647

**Published:** 2020-12-16

**Authors:** Rika Sakurai, Asa Inagaki, Yukitsugu Komazawa, Mari Kimata, Jun Goto

**Affiliations:** The University of Tokyo, Tokyo, Japan

## Abstract

Japan aims to enable older adults to remain at home in their familiar environment. However, the factors associated with daily life support for older adults who require medical and nursing care to remain at home are unclear. This study aimed to clarify the factors associated with daily life support for single older adults needing medical and nursing care to remain at home. Our participants were single older adults aged 65-94 years receiving medical and nursing care and their care providers. First, we analyzed records, which were written by care providers, regarding ten older adults who received medical and nursing care from 2014 to 2018. We categorized occurrences which exert single older adults’ life on change into six factors, such as gradual frailty and loss of a loved one. Then, to consider how they experience these factors, we conducted semi-structured interviews with three additional older adults who were single and received home visiting nursing care service in 2020. During this process, four multidisciplinary researchers discussed the factors associated with daily life support for single older adults; finally, three factors were derived. The first one pertained to health conditions: receiving sufficient medical and nursing care maintain older adults’ physical condition. The second related to the environment: maintaining social interactions (neighbors and friends). The third pertained to older adults’ values and meanings to remain at home. This study suggests that care providers focus on these three factors to help older adults who received medical and nursing care to remain at their home.

